# Characterization of a dual-action adulticidal and larvicidal interfering RNA pesticide targeting the *Shaker* gene of multiple disease vector mosquitoes

**DOI:** 10.1371/journal.pntd.0008479

**Published:** 2020-07-20

**Authors:** Keshava Mysore, Limb K. Hapairai, Longhua Sun, Ping Li, Chien-Wei Wang, Nicholas D. Scheel, Alexandra Lesnik, Jessica Igiede, Max P. Scheel, Na Wei, David W. Severson, Molly Duman-Scheel

**Affiliations:** 1 Indiana University School of Medicine, Department of Medical and Molecular Genetics, South Bend, Indiana, United States of America; 2 The University of Notre Dame Eck Institute for Global Health, Notre Dame, Indiana, United States of America; 3 The University of Notre Dame Department of Civil and Environmental Engineering and Earth Sciences, Notre Dame, Indiana, United States of America; 4 The University of Notre Dame Department of Biological Sciences, Notre Dame, Indiana, United States of America; 5 The University of the West Indies, Department of Life Sciences, St. Augustine, Trinidad, Trinidad and Tobago; International Atomic Energy Agency, AUSTRIA

## Abstract

The existing mosquito pesticide repertoire faces great challenges to sustainability, and new classes of pesticides are vitally needed to address established and emerging mosquito-borne infectious diseases. RNA interference- (RNAi-) based pesticides are emerging as a promising new biorational mosquito control strategy. In this investigation, we describe characterization of an interfering RNA pesticide (IRP) corresponding to the mosquito *Shaker (Sh)* gene, which encodes an evolutionarily conserved voltage-gated potassium channel subunit. Delivery of the IRP to *Aedes aegypti* adult mosquitoes in the form of siRNA that was injected or provided as an attractive toxic sugar bait (ATSB) led to *Sh* gene silencing that resulted in severe neural and behavioral defects and high levels of adult mortality. Likewise, when provided to *A*. *aegypti* larvae in the form of short hairpin RNA (shRNA) expressed in *Saccharomyces cerevisiae* (baker’s yeast) that had been formulated into a dried inactivated yeast tablet, the yeast IRP induced neural defects and larval death. Although the *Sh* IRP lacks a known target site in humans or other non-target organisms, conservation of the target site in the *Sh* genes of multiple mosquito species suggested that it may function as a biorational broad-range mosquito insecticide. In support of this, the *Sh* IRP induced both adult and larval mortality in treated *Aedes albopictus*, *Anopheles gambiae*, and *Culex quinquefasciatus* mosquitoes, but was not toxic to non-target arthropods. These studies indicated that IRPs targeting *Sh* could one day be used in integrated biorational mosquito control programs for the prevention of multiple mosquito-borne illnesses. The results of this investigation also suggest that the species-specificity of ATSB technology, a new paradigm for vector control, could be enhanced through the use of RNAi-based pesticides.

## Author summary

New classes of environmentally-safe pesticides are vitally needed to address established and emerging mosquito-borne infectious diseases. In this investigation, we describe characterization of an interfering RNA pesticide corresponding to the mosquito *Shaker* gene. Although the pesticide recognizes a conserved target site in the *Shaker* genes of multiple species of disease vector mosquitoes, it lacks a known target site in humans or other non-target organisms. The pesticide killed adult mosquitoes when it was microinjected or provided to adults as an attractive toxic sugar bait. The pesticide also induced high mortality rates when fed to larvae using a yeast-based expression and delivery system. These studies demonstrated that interfering RNA pesticides targeting the mosquito *Shaker* gene could one day be used for the biorational control of mosquitoes and the prevention of multiple mosquito-borne illnesses.

## Introduction

Although mosquito control is the primary means of preventing mosquito-borne illnesses, mosquito resistance to every class of chemical insecticides has been documented in many species across the globe [1], and the potential for adverse effects of insecticides on non-target species must be continuously monitored [2]. New insecticides and mosquito control strategies that pose low risks to human health and the environment are vitally needed to address established and emerging arthropod-borne infectious diseases [3]. RNAi, a regulatory pathway in eukaryotic cells that silences gene expression through production of siRNAs, is often used for functional characterization of insect genes in the laboratory [1]. We recently began a concerted effort to apply RNAi technology, which is attracting attention in the insect agricultural pest control community [4], to the field for mosquito control. To initiate these studies, we pursued screens that led to the identification of siRNAs that target genes required for mosquito larval viability [5–8]. Following discovery of these siRNAs, *S*. *cerevisiae* (baker’s yeast) was bioengineered to express shRNAs corresponding to the larvicidal siRNAs, creating a yeast expression and delivery system for larvicidal IRPs [5, 9, 10]. Subsequent work has prioritized characterization of yeast IRPs with target sites that are conserved in multiple species of disease vector mosquitoes. For example, recent studies demonstrated that yeast IRPs which recognize conserved sites in the mosquito *synaptotagmin* [7] and *semaphorin 1a* [8] genes can function as larvicides that kill *Aedes* (dengue, Zika, yellow fever and chikungunya vector), *Anopheles* (malaria vector), and *Culex* (West Nile and lymphatic filariasis vector) mosquitoes.

A subset of the siRNA larvicides identified in our recent screens corresponds to genes that are known to be required for both larval and adult viability in the genetic model insect *Drosophila melanogaster*. It was therefore hypothesized that these siRNAs could function as IRPs that target both mosquito adults and larvae. To evaluate this hypothesis, in this investigation we pursued characterization of an siRNA with a conserved target site in mosquito *Shaker (Sh)* genes. The *D*. *melanogaster Sh* gene, which encodes an evolutionarily conserved subunit of the voltage-gated potassium channel, is a well-characterized and historically important gene, as it was the first potassium channel gene to ever be cloned [11, 12]. Functioning channels containing the Sh subunit form a pore that carries type-A potassium currents [13]. Mutations in the *Drosophila Sh* gene [14, 15], as well as the mammalian ortholog *KCNA1* [16], result in hyperexcitability near axon branch points due to improper repolarization of neurons. These neural defects manifest behaviorally in uncontrolled movements [13]. For example, *Drosophila Sh* mutants exhibit aberrant movements that are most pronounced under ether anesthesia, when the mutant flies’ legs shake (hence the name *Shaker*, which describes the mutant phenotype) [17–19]. Even in the absence of anesthesia, *Sh* mutant fruit flies display uncoordinated walking behavior and are found to stand quivering on the bottom of the culture bottle [20]. Loss of *Sh* function can be lethal to fruit flies during the embryonic, larval, and adult stages [21, 22], suggesting that targeting this gene in mosquitoes could lead to mortality at multiple stages of the mosquito life cycle.

In this investigation, we demonstrate that silencing the *A*. *aegypti Sh* gene results in high rates of mortality in both adults and larvae. The mode of action for Sh IRPs is examined in the adult and larval nervous systems. A scalable ATSB-based delivery system for delivery of *Sh* IRPs to adult mosquitoes under simulated deployment conditions is examined, and a yeast-based system for delivery of *Sh* IRPs to larvae is developed and characterized. In addition to assessing *A*. *aegypti*, the activity of Sh.463 ATSBs and yeast are investigated in multiple species of disease vector mosquitoes, and the toxicity of these pesticides is also evaluated in several non-target arthropods species. The results of these studies suggest that IRPs targeting mosquito *Sh* genes may represent a new biorational intervention that can be used in integrated mosquito control programs targeting multiple species of disease vector mosquitoes.

## Methods

An overview of the experimental research plan is provided in S1 Fig.

Mosquito rearing: The following mosquitoes were used in this investigation: *A*. *aegypti* Liverpool-IB12 (LVP-IB12) strain mosquitoes, *A*. *albopictus* (obtained from BEI Resources, NIAID, NIH: *A*. *albopictus*, Strain Gainesville, MRA-804, contributed by Sandra A. Allan), *A*. *gambiae* G3 strain mosquitoes (obtained through BEI Resources, NIAID, NIH: *A*. *gambiae*, Strain G3, Eggs, MRA-112, contributed by Mark Q. Benedict), and *C*. *quinquefasciatus* (provided by Centers for Disease Control and Prevention for distribution by BEI Resources, NIAID, NIH: *C*. *quinquefasciatus*, Strain JHB, Eggs, NR-43025). These mosquito strains were reared as described previously [23] with the exception that adult females were fed using a Hemotek artificial membrane feeding system (Hemotek Limited, Blackburn, UK) to deliver sheep blood (purchased from HemoStat Laboratories, Dixon, CA). The insectary used for mosquito rearing was maintained at 26° C, ~80% relative humidity, and with a 12 hr dark/12 hr light cycle that included 1hr crepuscular periods at the beginning and end of each cycle.

Identification of siRNA #463: siRNA #463, which was not described previously, was assessed through siRNA larval soaking [5, 6] and adult microinjection (see below) studies. This sequence/gene was screened because the *D*. *melanogaster* ortholog is known to be required for larval viability [24], and the *A*. *aegypti* ortholog is known to be expressed throughout larval development (per Akbari et al. [25]). The larval soaking experiments were conducted using the Singh et al. [26] soaking protocol on first instar (L1) larvae as previously described [5, 6] using siRNAs purchased from Integrated DNA Technologies (Coralville, Iowa) that corresponded to the following target sequences: #463: 5’- AUUUAAAUUAUCUAGGCAUUCGAAA -3’ in *Shaker (AAEL000242)* and a control sequence that has not been identified in any of the mosquito species [27]: 5’-GAAGAGCACUGAUAGAUGUUAGCGU-3’. The soaking trial was performed in duplicate experiments conducted on 20 first instar larvae (40 larvae evaluated in total per control or experimental treatment) that were soaked in 0.5 μg/μl siRNA for four hours, then reared and assessed as described in the World Health Organization (WHO) larvicide testing guidelines [28]. A one-tailed Fisher’s exact test (SPSS software, IMB, Armonk, NY) was used for evaluation of soaking data. The Fisher’s exact test with a 0.05 significance level was selected for analysis of these data, and for several other statistical analyses performed in this investigation (see below), when assumptions for using a t-test were not met. Data from independent trials were analyzed both independently, as well as following combination of data from individual trials, with both analyses yielding similar outcomes; P values are reported for the combined data sets only in the interest of simplicity.

The adulticidal capacity of Sh.463 was evaluated through microinjection of adult female *A*. *aegypti* mosquitoes. Two replicate experiments were performed using a modification of an embryo microinjection protocol [29]. In these experiments, three-day old non-blood fed adult females/treatment were anesthetized with carbon dioxide gas and microinjected vertical to the body axis in the thoracic region with 250 nl of 9 μg/μl experimental or control siRNA. Following injection, adults were placed in a cage to recover, and adult mortality was assessed for the next week. Twenty individuals were injected per treatment for each replicate, and experiments to confirm each phenotype were repeated four times (80 adults injected in total for each control or experimental treatment). A one-tailed Fisher’s exact test was used for evaluation of adult microinjection data. Results from each individual trial were analyzed independently, as well as following combination of all trial data, generating similar outcomes in both cases (P values of the combined analyses are reported in the results). *A*. *albopictus*, *A*. *gambiae*, and *C*. *quinquefasciatus* susceptibility to Sh.463 siRNA was evaluated using this same protocol and data analysis plan, except that three biological replicate trials were performed, each on a total of 20 mosquitoes/treatment (60 adults injected per control or experimental treatment in total), and the dose was reduced to 150 nl of 6 μg/μl siRNA per adult for *A*. *gambiae* (which are smaller than *A*. *aegypti* adult mosquitoes) and increased to 400 nl of 12 μg/μl siRNA per adult for *C*. *quinquefasciatus* (which are larger than *A*. *aegypti* mosquitoes).

Adult ATSB simulated field trials: The pointed end of a 0.2 ml PCR tube (Eppendorf, Hauppauge, NY) was cut off with a razor blade, creating a 1 mm opening. A small piece of cotton (3–4 mg) was placed in the end of the tube to create a wick. 64 μl of 10% sucrose solution (in sterile DEPC-treated water) containing 0.5% of blue tracer dye (McCormick) alone or with 2.5 μg/μl of control or Sh.463 siRNA and was pipetted in the tube. The tube was capped and hung at the top of a 3.75 L cage (Berry Global, Evansville, IN) with the wick facing inward (down) to allow mosquitoes to feed from the wick [30]. For each control or experimental treatment, 25 4–5 day old adult females that had not blood fed and which had been sugar-starved for 48 hrs were permitted to feed for four hrs (beginning at dawn). Females that failed to feed or that were semi-engorged were discarded. Engorged mosquitoes were carefully collected as individuals and placed in *Drosophila* rearing vials. After 24 hrs, these individuals were fed with 10% sucrose solution (lacking siRNA), which was subsequently provided every 48 hrs. Behavioral phenotypes and mortality were scored daily for six days. Three biological replicate experiments were conducted, with 25 insects assessed per treatment (75 adult females in total assessed for each control or experimental treatment). The G-test of independence (SPSS software, IMB, Armonk, NY) was used to compare feeding rates among treatments and to examine if the proportions at one variable (fed) are the same for different values of the second variable (unfed); data on individual trials, as well as data combined from separate trials were analyzed, with similar results obtained in both cases and P values reported for the combined analyses in the interest of simplicity. Log-rank tests (SPSS software, IMB, Armonk, NY) were used to compare survival rates among the control (sugar bait alone or sugar bait with control siRNA) or experimental (sugar bait with Sh.463 siRNA) mosquitoes that had consumed the sugar meals provided.

Generation of yeast interfering RNA larvicide strains and yeast culturing: Custom DNA oligonucleotides encoding an shRNA expression cassette corresponding to the Sh.463 target sequence were purchased from Invitrogen Life Technologies (Carlsbad, CA) and used to generate stable transformant *S*. *cerevisiae* as previously described [5]. In summary, DNA encoding the Sh.463 hairpin was ligated downstream of the galactose-inducible *Gal1* promoter [31] and upstream of the *cyc1* terminator; the resulting *Gal1* promoter-Sh.463 shRNA-*cyc1* terminator expression cassette was inserted into the multiple cloning sites of the *pRS404* and *pRS406* integrating shuttle vectors [32], which bear *TRP1* and *URA3* selection markers, respectively. The resulting plasmids, which were marked by *TRP1 (pRS404*) and *URA3 (pRS406)*, facilitated chromosomal integration and selection of *S*. *cerevisiae CEN*.*PK* strain (genotype *MAT*a/α *ura3-52/ura3-52 trp1-289/trp1-289 leu2-3_112/leu2-3_112 his3* Δ*1/his3* Δ*1 MAL2-8C/MAL2-8C SUC2/SUC2* [33]) yeast through growth on synthetic complete media that lacked tryptophan and uracil. PCR and sequencing confirmed integration of the Sh.463 expression cassettes at both loci. This strain, hereafter referred to as Sh.463 yeast IRP, as well as a stably transformed control shRNA expression strain constructed in a previous study [5], were used in these investigations.

shRNA expression from these strains was confirmed in each of two biological replicate trials for each strain (control or Sh.463) in which total RNA was extracted using TRIzol Reagent according to the manufacturer’s instructions (Invitrogen, Carlsbad, CA) from 5.6 mg of pelleted yeast taken from cultures that had been prepared as described [5]. cDNA was prepared according to the instructions provided in the High Capacity RNA to cDNA Kit (Applied Biosystems, Foster City, CA). 1/100 of the resulting cDNA was used as template for PCR amplifications performed with Clontech Labs 3P TaKaRa Taq DNA Polymerase (Clontech Laboratories, Mountain View, CA) per the manufacturer’s instructions in conjunction with the following primer sets: Control shRNA Forward 5’-ACGCTAACATCTATCAGTGC-3’ (specific to control shRNA) or Sh.463 shRNA Forward 5’-TCGAATGCCTAGATAATTTAA-3’ (specific to Sh.463 shRNA) and Reverse primer 5’-TCCTTCCTTTTCGGTTAGAGC-3’ (which corresponds to the terminator in both the control and Sh.463 strains). For larvicide assays, dried inactivated yeast interfering RNA tablets were prepared from these strains using a previously published protocol [9].

Laboratory larvicide assays: Laboratory larvicide trials that conformed to the WHO larvicide testing guidelines [28] were conducted in the insectary as described [6]. In summary, for each of three biological replicate trials, 20 first instar larvae were placed in 50 ml of distilled water in each of three 500 ml replicate containers/condition. In each container, the 20 larvae were provided with a single control or Sh.463 yeast tablet at the beginning of the trial, and this was sufficient to permit the larvae to feed on the yeast *ad libitum* throughout the experiment. When larvae reached the fourth instar (L4), 150 μl of 6% w/v liver powder (MP Biomedicals) mixed in distilled water was added to each container as a larval dietary supplement as described [9]. Larval mortality was assessed throughout the trial period. At the conclusion of the trial, the percentages of larval mortality were transformed using arcsine transformation as described [28]. For *A*. *aegypti*, data from three biological replicate trials, each with three replicate containers bearing 20 larvae per treatment (nine containers total per control or experimental treatment, with a total of 180 larvae evaluated per treatment) were assessed with a paired one-tailed t-test (SPSS software, IMB, Armonk, NY). For *A*. *albopictus*, *A*. *gambiae*, and *C*. *quinquefasciatus*, data from three trials, each with four containers (12 containers total per control or experimental treatment, with a total of 240 larvae evaluated per treatment) were evaluated in a similar manner.

Dose-response curves were produced as described [5] following the conduction of three biological replicate trials. For each trial, three larvicide-treated replicate containers for each of 12 control or Sh.463 yeast dosages were analyzed, or in total nine replicate containers per dosage, each with 20 larvae (180 total larvae assessed per control or Sh.463 dosage). For generation of different dosages of Sh.463 IRP yeast, the larvicidal yeast culture was mixed with the control shRNA yeast culture in various proportions. For analysis of dose-response curve data, Abbot’s formula was used to account for <2% mortality in control larvae as discussed previously [28], and replicate data were pooled for analysis. LD_50_ values with 95% confidence intervals were determined through use of SPSS 25 software (IMB, Armonk, NY) and log dosage-probit mortality as described [5, 6].

Semi-field larvicide trials: Semi-field larvicide trials were performed outdoors in a rooftop laboratory in Notre Dame, IN during May and June 2019. These assays, which were performed in accordance with the WHO larvicide testing guidelines [28], were conducted using LVP-IB12 strain *A*. *aegypti* mosquitoes as previously described [7, 8]. 20 L1 larvae, 3.7 L of distilled water (water height of 10 cm), and one Sh.463 or control yeast tablet that had been prepared as described [9] were placed in each 10 L replicate container (diameter = 23 cm, height = 25 cm). Each replicate container was covered with mesh and placed in a screened SansBug 1-Person Free-Standing Pop-Up Mosquito-Net tent (Hakuna Matata Tents, Ontario, Canada) that was placed underneath an overhang. The tent mesh (472 openings per centimeter) provided a second level of enclosure to prevent mosquito escape and the entrance of macrobiota into the test site. Three biological replicate trials were completed, the first with six, the second with five, and the third with three replicate containers per condition (14 total containers assessed per treatment, and 280 larvae per control or experimental treatment assessed in total). The percentages of larval mortality were transformed using arcsine transformation, and data from multiple replicate experiments were assessed with a paired one-tailed t-test. During the testing period, temperatures ranged from 9° C to 35° C. The mean daytime temperature and nighttime temperatures during this trial period were 23.5±5° C and 19±4° C, respectively, while humidity levels averaged 75±15%.

Whole mount *in situ* hybridization and immunohistochemistry: The Patel [34] protocol was used for synthesis of a riboprobe corresponding to the *Aae Sh* gene, which was used for *in situ* hybridization experiments that were performed on adult and L4 larval brains as described [35]. For adults, four biological replicate experiments were conducted, each using brains from 20 adult females that had been microinjected with control or 20 adult females that had been injected with Sh.463 siRNAs as described above. For larvae, three biological replicate experiments were performed, each using the brains of 20 larvae per treatment that had been fed either Sh.463 or control yeast tablets according to the laboratory larvicide trial protocol described above. Processed brain tissues were mounted and imaged with a Zeiss Axioimager (Carl Zeiss Microscopy, LLC, Thornwood, NY) equipped with a Spot Flex camera (Diagnostic Instruments, Inc. Sterling Heights, MI). Following imaging, mean gray values (average signal intensity over the selected area) were calculated using FIJI ImageJ software [36], which facilitated quantification of digoxigenin-labeled *Aae Sh* transcript signals in the brains of control or Sh.463-treated mosquitoes as described [37]. Transcript level data from the three larval or adult biological replicate experiments were combined and analyzed with a t-test (two-tailed, equal variance). P values for these combined data analyses are reported in the results section. However, these statistical results were further confirmed through independent analysis of trial data from each individual repeat trial, which yielded similar outcomes.

Immunohistochemical staining experiments were performed on the brains of control or Sh.463-treated L4 or adult animals as described [38, 39]. mAb nc82 anti-Bruchpilot (40) (DSHB Hybridoma Product nc82, which was deposited by E. Buchner to the DSHB) and TO-PRO-3 iodide (Molecular Probes, Eugene, OR) were used in the experiments, which were conducted in triplicate using brains from 20 L4 larvae or adults per treatment. After completion of immunohistochemical processing, tissues were mounted and imaged with a Zeiss 710 confocal microscope and Zen software. The images were analyzed with FIJI ImageJ [36] and Adobe Photoshop CC 2018 software, which facilitated quantification of mean gray values (average signal intensity over the selected area) that were calculated as described [37]. Data from the control and IRP treatment groups were combined and statistically analyzed with a t-test (two-tailed, equal variance). P values for these combined data analyses are reported in the results section, but these statistical results were further confirmed through independent analysis of trial data from each individual repeat trial, which yielded similar outcomes.

*D*. *melanogaster* toxicity studies: The survival of Oregon R [24] *D*. *melanogaster* larvae that fed on Sh.463 or control interfering RNA yeast was evaluated as described [8]. In summary, for each assay, a pellet of dried inactivated Sh.463 or control interfering RNA yeast prepared as described above was resuspended in 200 ul of distilled water and 10 ul of McCormick’s red food dye. In each biological replicate assay, the Sh.463 or control yeast-food mixture was fed to 20 first instar larvae per treatment that had been placed in the vial of food. Yeast consumption was confirmed through observation of red food dye in the larval guts during the entire experimental period. These toxicity studies were performed at 22° C under ambient laboratory illumination (12 hr light/12 hr dark). The number of adults that emerged from each replicate tube was recorded as a measurement of survival. Seven biological replicate experiments, each with 20 larvae per control or experimental treatment (140 larvae per treatment) were performed. Data combined from these trials were evaluated with a two-tailed Fisher’s exact test. Data from each repeat trial were also analyzed independently in this manner, and similar P values were obtained.

For evaluation of adults, 20 females per treatment were placed in a 100x150mm petri dish with 32 μl of 10% sucrose solution containing 0.5% of blue tracer dye (McCormick) alone or with 2.5 μg/μl of control or Sh.463 siRNA. Four 8 μl droplets of control or experimental solution were placed at the bottom of a petri dish. Adult flies fed overnight before being transferred into vials containing fruit fly rearing media. These toxicity studies were performed at 22° C under ambient laboratory illumination (12 hr light/12 hr dark). Mortality was recorded daily for six days. Data from two biological replicate experiments (40 females in total assessed per control or Sh.463 treatment) were collected. Data were analyzed using a G-test of independence, which yielded similar P values for individual trials or following combination of trials from multiple repeat trials.

*Daphnia* toxicity assays: *Daphnia magna* and *Daphnia pulex* were purchased from Carolina Biologicals (Burlington, NC) and evaluated as described [8]. In summary, 20 adults per treatment of each *Daphnia* species were reared on Sh.463 or control yeast in each biological replicate trial. The toxicity trials were performed at 22° C and under ambient laboratory illumination (12 hr light/12 hr dark) in COMBO medium containing 0.0001% sodium selenium [41]. For each replicate assay, a single Sh.463 or control yeast tablet was dissolved in 50 mL of distilled water and fed to the *Daphnia* over the course of five days (10 ml of solution/day). *Daphnia* survival was evaluated daily during a 10 day trial period, and survival data from six biological replicate trials (120 animals assessed in total per control or Sh.463 treatment) were combined and analyzed using a two-tailed Fisher’s exact test. Results from each individual trial were also assessed independently in this same manner, yielding comparable P values.

*Tribolium castaneum* toxicity studies: *T*. *castaneum* obtained from Carolina Biologicals (Burlington, NC) were reared according to the instructions provided. 10 g of an 8:8:1:1 mixture of white flour, brown flour, nutritional yeast (all provided by Carolina Biologicals), and control or Sh.463 yeast (prepared through grinding a yeast tablet into powder) was placed in a rearing vial (provided by Carolina Biologicals). 20 newly hatched *T*. *castaneum* larvae were added to each control or experimental tube and monitored throughout their development into adults (~62 days). The number of adults eclosed per replicate tube was observed and recorded for survival analyses. Data from six biological replicate trials (with 120 beetles assessed in total per control or Sh.463 treatment) were analyzed using a two-tailed Fisher’s exact text. Data from each trial were analyzed both independently, as well as following combination of the data from all six trials, generating similar P values.

## Results and discussion

### Discovery of Sh.463 siRNA, a dual-action adulticidal/larvicidal mosquito IRP

**Fig 1:**
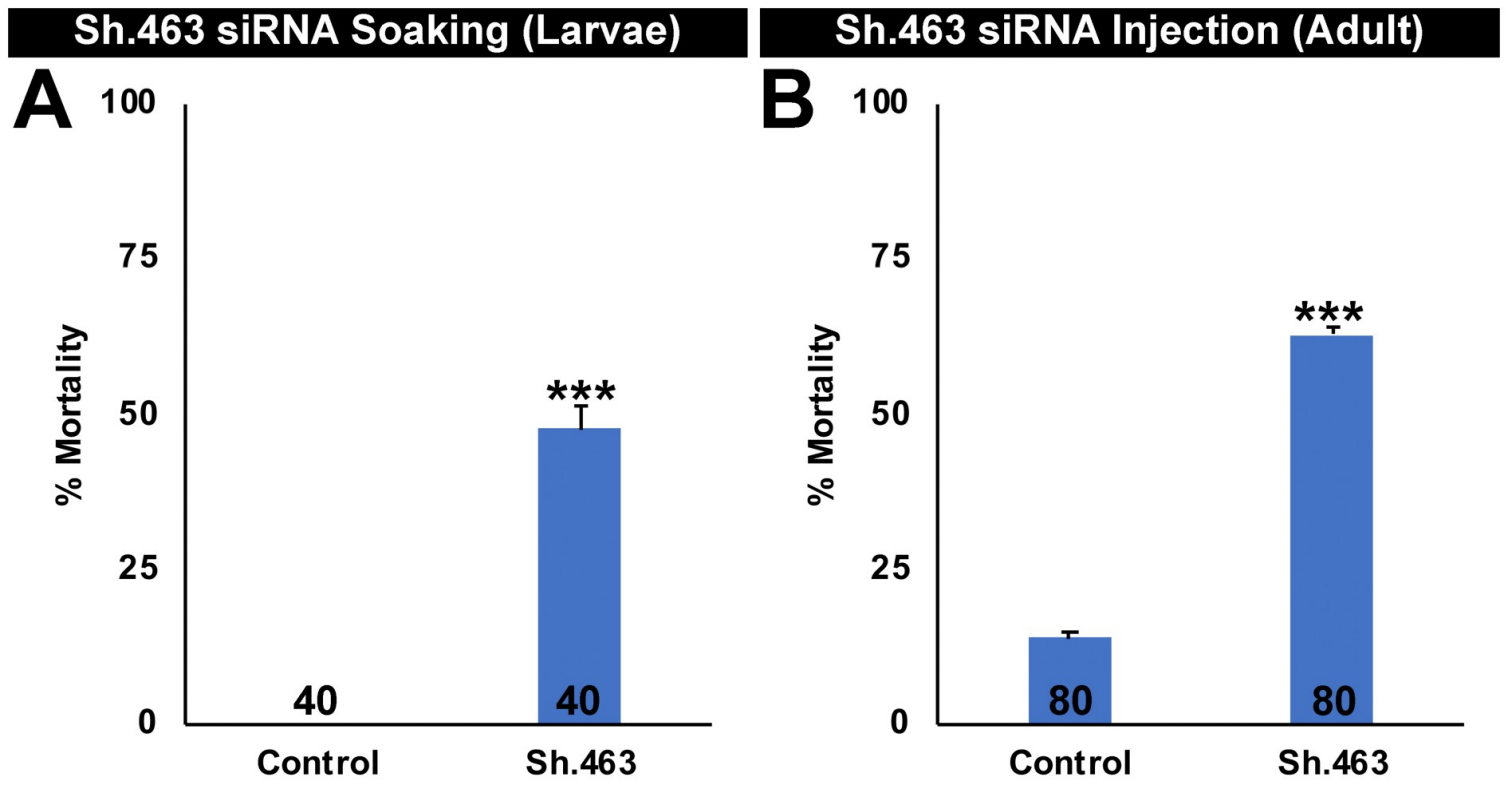
Sh.463 siRNA has larvicidal and adulticidal activity in *A*. *aegypti*. Significant larval mortality was observed after L1 larvae were soaked in 0.5 ug/ul Sh.463 siRNA (A). Significant adult mortality was observed after adult females were injected with 250 nl of 9 μg/μl Sh.463 siRNA (B). Data from Sh.463 vs. control siRNA-treated mosquitoes are represented here as mean percentage mortality; *** = P<0.001; error bars denote standard errors of the mean (SEM). The samples sizes for each treatment are noted on the columns in each graph in this figure and in all subsequent figures.

Sh.463 siRNA corresponds to a target sequence in exon nine of *A*. *aegypti Shaker (Aae Sh)* that is conserved in multiple species of disease vector mosquitoes, but not non-target organisms (S1 Table). As discussed above, published reports on *D*. *melanogaster Sh* mutant characterization [21, 22], combined with *A*. *aegypti* developmental transcriptome data [25] that confirmed expression of this gene throughout larval development and in adults led to the hypothesis that Sh.463 siRNA would induce mortality in *A*. *aegypti* larvae and adults. In larval soaking experiments, Sh.463 siRNA induced 48±4% larval mortality when first instar larvae were soaked for four hours in 0.5 ug/ul Sh.463 siRNA (Fig 1A; P = 1.3X10^-8^ vs. control siRNA treatment, one-tailed Fisher’s exact test; control = 0±0% mortality). Furthermore, 63±1% mortality was observed in *A*. *aegypti* adult females that were microinjected in the thorax with Sh.463 siRNA (Fig 1B; P = 9.34X10^-11^ vs. control siRNA treatment, one-tailed Fisher’s exact test; control = 16±1% mortality). The results of these studies demonstrated that Sh.463 siRNA has both larvicidal and adulticidal activity in *A*. *aegypti*. These data indicate that *A*. *aegypti*, like *D*. *melanogaster* [21, 22], requires *Sh* function for survival at multiple stages of the insect life cycle.

**Fig 2:**
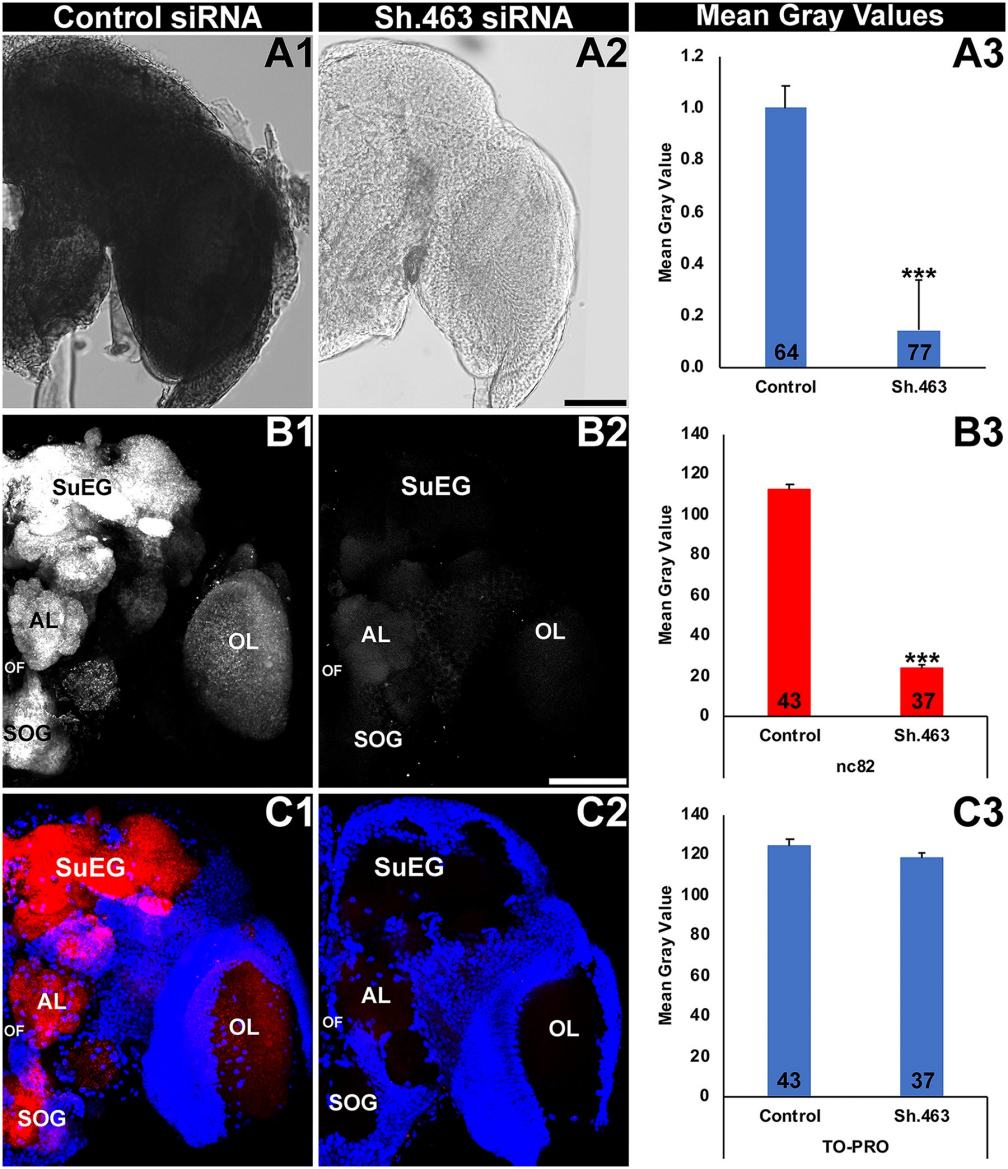
siRNA-mediated silencing of *Aae Sh* results in neural defects in *A*. *aegypti* adults. The high levels of *Aae Sh* expression detected throughout the *A*. *aegypti* adult female brain (a control siRNA-treated brain is shown in A1) were significantly reduced in adults injected with Sh.463 siRNA (A2). Adult brains were labeled with mAbnc82 (white in B1, B2; red in C1, C2), which labels synaptic active zones, and the nuclear stain TO-PRO (blue in C1, C2). nc82 levels were significantly reduced (B3) in the synaptic neuropil of adult females injected with Sh.463 siRNA (B2, C2; compare to white staining of control siRNA-injected brain in B1/red staining in C1). In A3, B3, and C3, data are represented as average mean gray values, and error bars denote SEM; *** = P<0.001 when compared with control siRNA-injected mosquitoes. Representative adult brains are oriented dorsal upward in this figure. **AL:** antennal lobe; **OL:** optic lobe; **SOG:** sub-esophageal ganglion; **SuEG:** supra-esophageal ganglion. Scale Bar = 100 μm.

As discussed above, *Sh* encodes an evolutionarily conserved subunit of a voltage-gated potassium channel that regulates neural activity in invertebrate and vertebrate organisms [11, 12]. It was therefore hypothesized that silencing of *Aae Sh* expression by Sh.463 siRNA would impact neural activity in *A*. *aegypti*. First, silencing of *Aae Sh* transcripts by Sh.463 siRNA was confirmed in the *A*. *aegypti* adult brain. *Aae Sh* is normally expressed broadly throughout the adult brain (Fig 2A1). A significant reduction in *Sh* transcripts was observed in the brains of adults injected with Sh.463 siRNA (Fig 2A2; 87% reduction with respect to control siRNA-treated brains, P = 4.3X10^-131^, t-test: two-tailed, equal variance). To evaluate if neural activity is compromised in Sh.463-treated adults, nc82 antibody staining, which reveals expression of Bruchpilot, a marker of active neural synapses [40], was assessed following Sh.463 siRNA injection. Although neural density (as shown through quantification of TO-PRO nuclear staining, Fig 2C3) was not significantly different (P>0.05, t-test: two-tailed, equal variance) in Sh.463 (Fig 2C2) vs. control-treated brains (Fig 2C1), nc82 levels were found to be reduced by 79% (Fig 2B3, P = 1.02X10^-46^, t-test: two-tailed, equal variance) in the brains of adults injected with Sh.463 siRNA (Fig 2B2 and 2C2) with respect to control-treated brains (Fig 2B1 and 2C1). These experiments indicate that the mode of action for Sh.463 siRNA in adult *A*. *aegypti* is through silencing of the *Aae Sh* gene and disruption of neural function.

Given that Sh.463 siRNA can function as an adulticide, identification of a field-appropriate mechanism for delivery of this IRP to mosquitoes could be beneficial. Unfortunately, effective delivery systems for topical application of IRPs to insects have not yet been developed. Attractive toxic sugar baits (ATSBs), a new paradigm for vector control [42], exploit the sugar feeding behavior of female and male mosquitoes that are lured to feed on a sugar source containing an insecticide. ATSBs, which are delivered through bait stations or as sprays, can be used both indoors and outdoors [42, 43]. Successful field trials in which significant reductions in disease vector mosquitoes were observed [42–44] indicate that this technology will significantly advance integrated mosquito control programs. We therefore evaluated a sugar bait IRP delivery system in simulated field studies.

**Fig 3:**
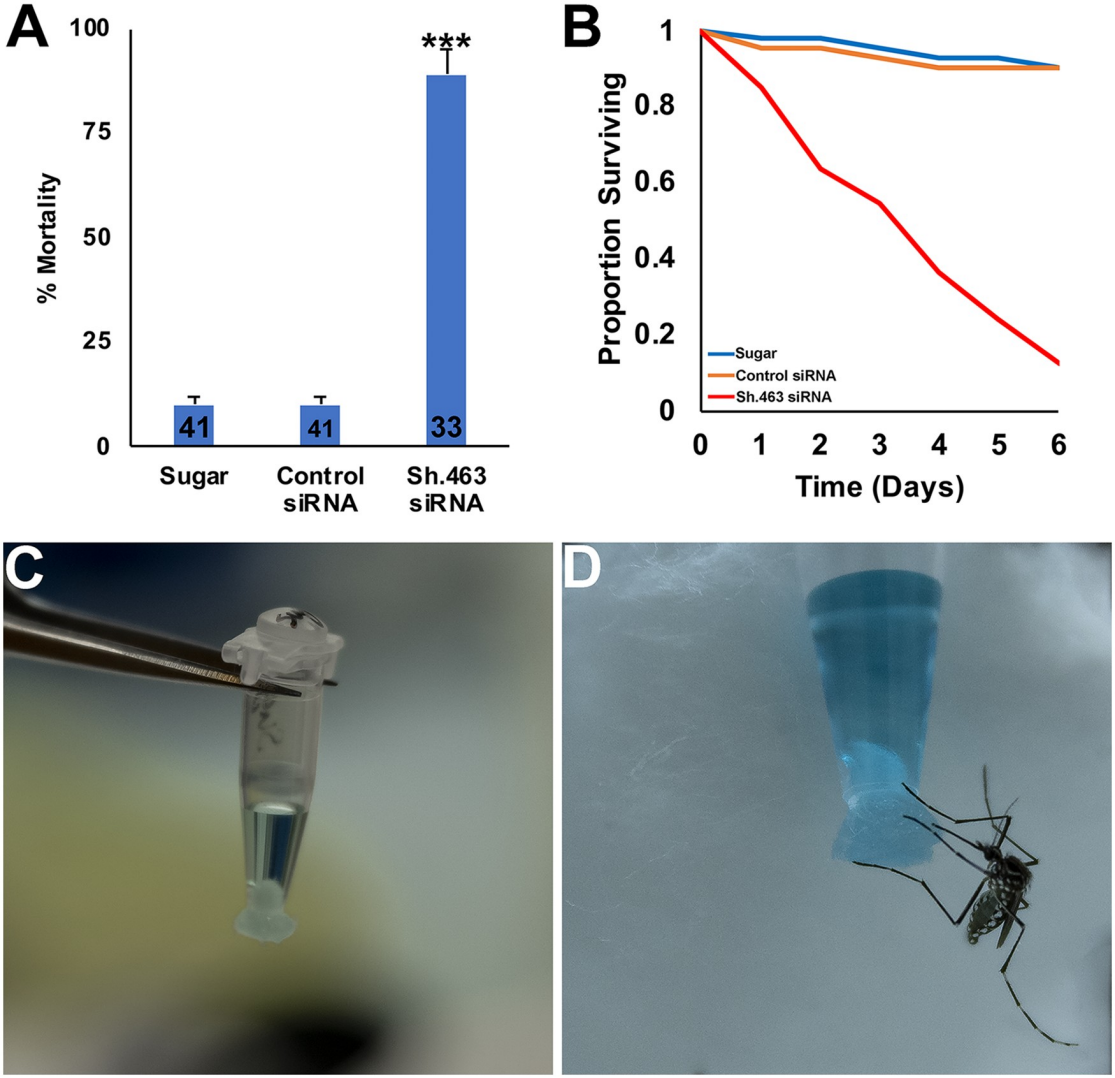
Delivery of Sh.463 siRNA as an ATSB induces high adult mortality. A. Sh.463 siRNA consumed as an ATSB by adult females induced significantly high adult mortality in comparison to sugar bait alone (Sugar) or sugar bait with control siRNA (Control). *** = P<0.001 vs. both Sugar and Control, which were not significantly different from each other; error bars represent SEM. B. A survival curve for adults that fed on sugar bait, control siRNA sugar bait, or Sh.463 sugar bait is shown. C. The delivery system for sugar bait marked with blue tracer dye is shown. D. An *A*. *aegypti* female feeding from the sugar bait station is observed.

The IRP delivery system used in these simulated field studies, which was modified from the procedure of Coy et al. [30], consisted of a wick that was created using a small tube with an opening that was plugged by a small piece of cotton (Fig 3C). The tube was then filled with sugar bait, which consisted of 10% sucrose marked with blue tracer dye that was used to track mosquito feeding (Fig 3D). Adult female mosquitoes were allowed to feed on sugar bait alone, sugar bait with control siRNA, or sugar bait with Sh.463 siRNA. From a total of 75 female mosquitoes subjected to each treatment (25 per control or Sh.463 treatment in each of three replicate experiments), 55±2% were observed to have fed on sugar bait, 55±2% on sugar bait containing control siRNA, and 44±6% on Sh.463 ATSB. Mosquito feeding rates among the treatments were not significantly different (P>0.05, G-test). Although 10±2% mortality was observed in mosquitoes fed with sugar bait alone, and 10±2% mortality was observed following consumption of sugar bait containing control siRNA (Fig 3A and 3B), 89±6% of mosquitoes that fed on Sh.463 ATSB died (Fig 3A; P<0.001 compared to sugar, P<0.001 compared to sugar with control siRNA, log-rank test). These mortality rates are higher than those observed following injection of siRNA into adult mosquitoes (Fig 1B), presumably because the average dose delivered in the ATSB studies was higher (~12 μg siRNA) than that delivered through microinjection (~2.25 μg per mosquito). As illustrated by the survival curve shown in Fig 3B, Sh.463 ATSB-treated adults died over the course of six days, with the time of death spread fairly evenly over the six day trial period. An interesting phenotype was observed in all of the Sh.463-treated mosquitoes (n = 33 individuals combined from three biological replicate experiments), including the 11±6% of mosquitoes that survived Sh.463 ATSB treatments (Fig 3A). The mosquitoes were found to have uncoordinated walking behavior, often falling and not venturing beyond the bottom of the vial in which they were housed (S1 Video). The mosquitoes did not fly, and the legs of these mosquitoes often shook (S1 Video). Ganetzky and Wu [20] described adult fruit flies bearing the *Sh*^*16*^ mutation in a similar manner, indicating that the flies displayed uncoordinated walking behavior and stood quivering on the bottom of the culture bottle. Although a small percentage of Sh.463-treated mosquitoes recover and survive in a laboratory setting (Fig 3A), it is anticipated that these individuals, all of which displayed this behavioral phenotype, would also die in the wild. This could bring the mortality rates of *A*. *aegypti* mosquitoes treated with Sh.463 ATSB to 100% in the field. We hope to evaluate this in *A*. *aegypti* field trials in the future, as several studies suggest that ATSBs are an effective means of controlling *A*. *aegypti* [44, 45].

Delivery of Sh.463 through sugar baits could significantly enhance existing ATSB technology. Although sugar baits facilitate targeted delivery of a variety of pesticides, pesticide resistance is still of concern [46]. The development of new classes of pesticides, such as IRPs, that can be delivered as ATSBs may therefore be beneficial. IRPs could also enhance the species-specificity of ATSBs. For example, the boric acid and garlic oil insecticides used in many ATSBs are not specific to mosquitoes [46]. Despite the addition of protective barriers to bait stations and efforts to limit ATSB applications to non-flowering vegetation [42, 43], it is still difficult to completely eliminate risks to pollinators and other non-target organisms. IRPs, which appear to have a desirable safety profile [47], particularly when compared to conventional pesticides, could therefore make ATSBs more species-specific.

**Fig 4:**
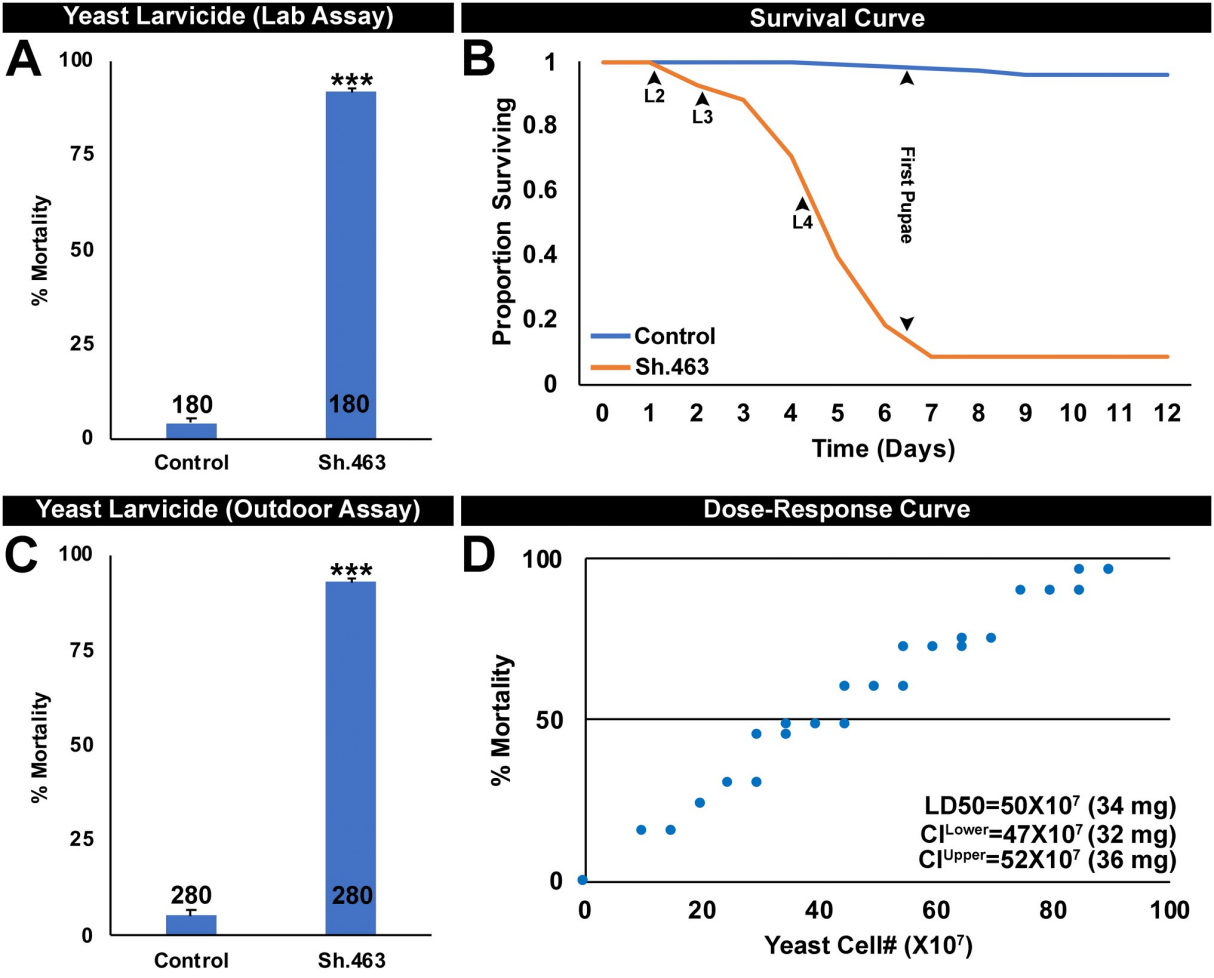
Sh.463 yeast IRP induces *A*. *aegypti* larval death. *A*. *aegypti* larval consumption of inactivated dried Sh.463 yeast IRP tablets induced significant larval mortality in laboratory (A) and outdoor semi-field (C) larvicide trials. In panels A and C, data are represented as mean percentage mortality; error bars represent SEM, and *** = P<0.001 in comparison to control yeast IRP-treated larvae. B. Consumption of inactivated dried Sh.463 yeast larvicide tablets induced larval mortality in the fourth instar (L4, days 4–6; compare to larvae fed with control yeast IRP that survived and pupariated). D. A dose-response curve depicting the dosage of Sh.463 yeast vs. the percentage mortality of *A*. *aegypti* larvae is shown; LD_50_ = 34 mg.

In recent studies, we have engineered *S*. *cerevisiae* to express shRNAs corresponding to larvicidal siRNAs identified in larval lethality screens [5–8, 10]. Yeast cultured from these strains can be heat-inactivated and dried into a tablet formulation that can be used as a larvicide [5]. In an effort to add to the growing arsenal of larvicidal yeast IRPs, stably transformed *S*. *cerevisiae* expressing shRNA corresponding to Sh.463 siRNA (hereafter referred to as Sh.463 yeast) was generated. Total RNA was extracted from the Sh.463 yeast strain, as well as from the control yeast strain, and used for preparation of cDNA, permitting PCR verification that Sh.463 or control shRNAs were expressed in the two recombinant strains (S2 Fig). Dried inactivated yeast tablets prepared from the Sh.463 strain were found to induce 92±1% mortality [Fig 4A; P = 4.1X10^-19^ vs. control yeast interfering RNA treatment, paired one-tailed t-test (control = 4±1% mortality); LD_50_ = 34 mg, Fig 4D] in *A*. *aegypti* larvae. Larvae treated with Sh.463 yeast beginning in L1 died as L4 larvae (Fig 4B), a time that is consistent with the onset of death observed in *D*. *melanogaster* larval mutants, which died late in larval development or as early pupae [22]. It should be noted that the larval mortality rates observed following continuous larval consumption of Sh.463 yeast, from the first through fourth instars (Fig 4B), are much higher than those observed following brief four hour soaking treatments of L1 larvae (Fig 1A). Similar results have been observed in recent studies [5–8, 10].

**Fig 5:**
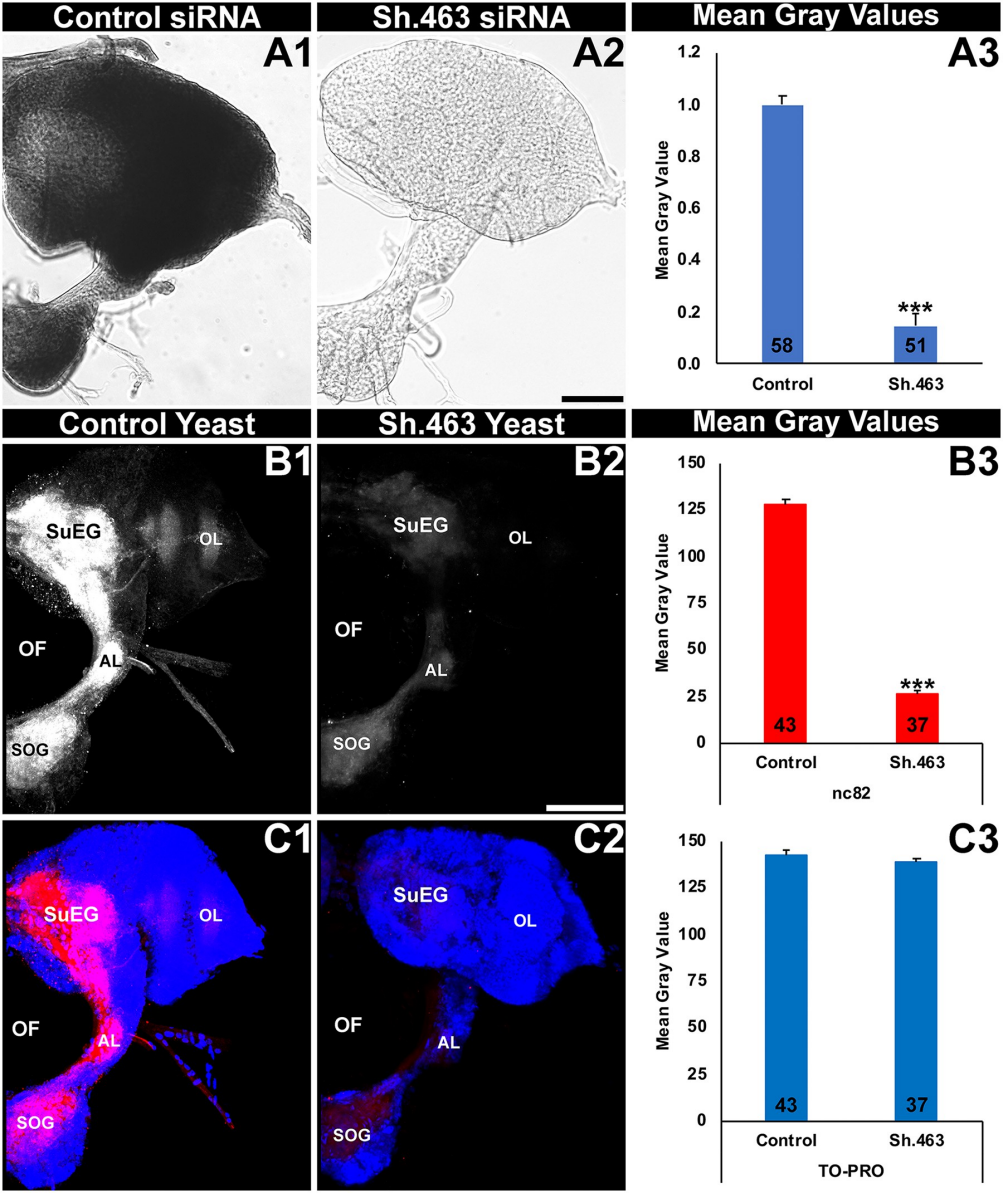
Sh.463 yeast induces neural defects in *A*. *aegypti* larvae. High levels of *Aae Sh* expression detected throughout the *A*. *aegypti* L4 larval brain (the brain from a control yeast-treated larva is shown in A1) are significantly reduced (A3) in the L4 brain of larvae fed with Sh.463 yeast (A2). Mean gray value analysis results are displayed in A3. Larval brains were labeled with mAbnc82 (white in B1, B2; red in C1, C2) and TO-PRO (blue in C1, C2). nc82 levels were significantly reduced (B3) in the synaptic neuropil of larvae fed with Sh.463 yeast (B2, C2; compare to control-treated brain in B1, C1). In B3 and C3, data are represented as average mean gray values. In A3, B3, and C3, error bars denote SEM, and *** = P<0.001 when compared with brains from control yeast-treated larvae. Representative adult brains are oriented dorsal upward in this figure. **AL:** antennal lobe; **OL:** optic lobe; **SOG:** sub-esophageal ganglion; **SuEG:** supra-esophageal ganglion. Scale Bar = 100 μm.

To confirm that the mode of action for Sh.463 yeast IRP treatment is through silencing of the *Aae Sh* gene, *Sh* transcript levels were assessed in the *A*. *aegypti* L4 brain. As observed in adults (Fig 2A1), in control yeast IRP-treated animals, *Sh* transcripts are expressed broadly in the early L4 larval brain (Fig 5A1). An 85±1% reduction in *Sh* transcripts (Fig 5A3, P = 4.45X10^-127^, t-test: two-tailed equal variance) is observed in the brains of Sh.463 yeast-treated larvae (Fig 5A2) that were harvested in early L4 just prior to the time that treated animals die (Fig 4B), indicating that the time of death coincided with silencing of *Sh* transcript levels. As observed in adults (Fig 2), this loss of *Sh* transcripts coincided with significant loss of nc82 expression (P = 9.75X10^-51^, t-test: two-tailed, equal variance) in the brains of Sh.463 yeast-treated larvae (Fig 5B2 and 5C2 vs. control in Fig 5B1 and 5C1), but no significant differences (Fig 5C3, P>0.05, t-test: two-tailed, equal variance) were observed in TO-PRO nuclear staining levels among Sh.463-treated (Fig 5C2) or control-treated brains (Fig 5C1). These data indicate that neural activity, but not neural density, is compromised in larvae treated with Sh.463 yeast. This observed disruption of neural activity in the L4 nervous system coincided with the timing of larval death (Fig 4B). These experiments, combined with our analyses of adults (Figs 2 and 3), demonstrated that Sh function in the nervous system is required at multiple stages of the *A*. *aegypti* life cycle.

Finally, in preparation for future field studies, semi-field evaluation of Sh.463 yeast activity was pursued in an outdoor roof top laboratory in Notre Dame, IN. 93±1% larval death was observed in Sh.463-treated containers (Fig 4C; P = 3.11X10^-17^ vs. control, paired one-tailed t-test; control = 5±1% mortality). These results indicated that activity of Sh.463, like that of several other IRP yeast larvicides that have been constructed in our laboratory [7, 8], is retained during exposure to outdoor conditions and temperatures that ranged from 9° C to 35° C during the summertime testing period. These studies, combined with previous studies in which yeast IRP larvicides were shown to function in different types of water [7, 8], in different sizes of containers bearing 0.05–26 L of water [5, 7, 8], and with different numbers and densities of *A*. *aegypti* larvae [5, 7, 8], indicate that yeast IRPs may represent a new larvicidal intervention that could prove useful in integrated mosquito control programs. In ongoing investigations, we are working to scale yeast production to commercial levels. We are also working to develop long-lasting formulations with residual activities that extend beyond two weeks, the residual activity of our present dried tablet formulation [5]. In previous studies, we demonstrated that the ~10% of larvae that survive yeast IRP treatment are not resistant to yeast IRPs, but appear to be eating dead or dying larvae in the containers rather than yeast; all larvae die when treated with yeast IRPs and reared individually [7, 8]. By continuing to grow the arsenal of yeast IRPs, we are constantly developing alternative pesticides that could be used in the event that resistance develops to one specific yeast IRP.

### Sh.463 IRPs function as broad-range mosquito insecticides but are not toxic to non-target arthropods

**Fig 6:**
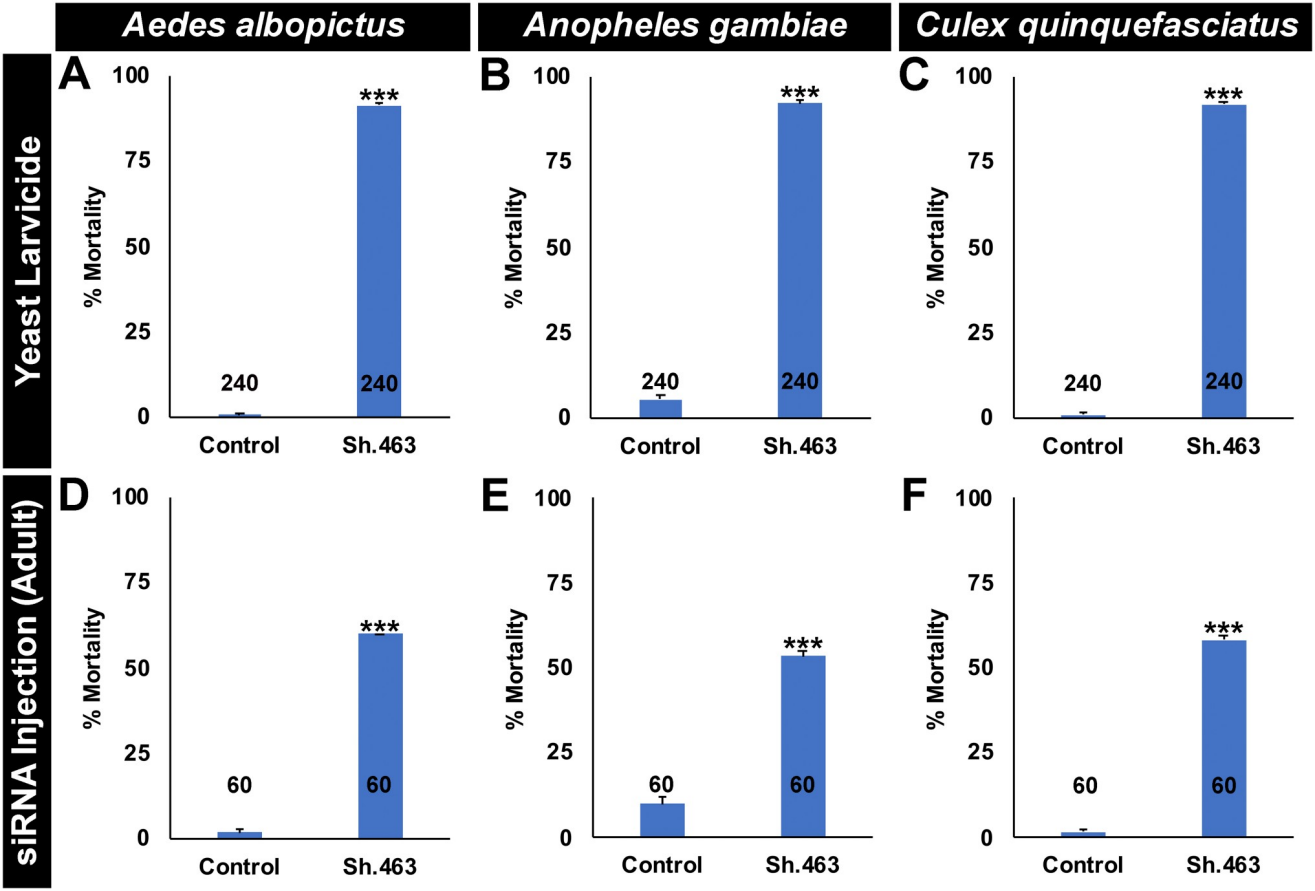
Sh.463 is a broad-range mosquito pesticide. Consumption of Sh.463 IRP yeast (A-C) induces high levels of mortality in *A*. *albopictus* (A), *A*. *gambiae* (B), and *C*. *quinquefasciatus* (C) larvae. Injection of Sh.463 siRNA induces mortality in *A*. *albopictus* (D), *A*. *gambiae* (E), and *C*. *quinquefasciatus* (F) adult females (see Methods for dosage information). The data are represented as mean mosquito mortality, and error bars denote SEM. *** = P<0.001 when compared with control-treated mosquitoes.

The target site of Sh.463 IRPs is conserved in *A*. *albopictus*, *C*. *quinquefasciatus*, and multiple species of *Anopheles* malaria vector mosquitoes, but not in the sequenced genomes of other insects, humans, or other non-target organisms (S1 Table). We recently identified IRPs with conserved target sites in mosquito *synaptotagmin* and *semaphorin 1a* genes that kill *Aedes*, *Culex*, and *Anopheles* larvae [7, 8]. We therefore anticipated that Sh.463 yeast could function as a broad-range mosquito IRP. As predicted, Sh.463 yeast treatments resulted in 91±1% larval mortality in *A*. *albopictus* (Fig 6A; P = 2.95X10^-24^ vs. control yeast treatment, paired one-tailed t-test; control = 0±1% mortality), 92±1% larval mortality in *C*. *quinquefasciatus* (Fig 6C; P = 9.15X10^-23^ vs. control yeast treatment, paired one-tailed t-test; control = 5±1% mortality), and 92±1% larval mortality in *A*. *gambiae* larvae (Fig 6B; P = 1.71X10^-18^ vs. control yeast treatment, paired one-tailed t-test; control = 5±1% mortality). Likewise, microinjection of Sh.463 siRNA resulted in 60±0% *A*. *albopictus* adult mortality (Fig 6D; P = 1.48X10^-24^ vs. control siRNA treatment, one-tailed Fisher’s exact test; control = 2±3% mortality), 58±1% *C*. *quinquefasciatus* adult mortality (Fig 6F; P = 4.58X10^-23^ vs. control siRNA treatment, one-tailed Fisher’s exact test; control = 5±4% mortality), and 53±1% *A*. *gambiae* adult mortality (Fig 6E; P = 1.92X10^-7^ vs. control siRNA treatment, one-tailed Fisher’s exact test; control = 10±2% mortality). These mortality levels are consistent with mortality levels observed in microinjected *A*. *aegypti* adult females (Fig 1B). These data support the hypothesis that Sh.463 IRPs function as broad-based mosquito larvicides and adulticides.

**Fig 7:**
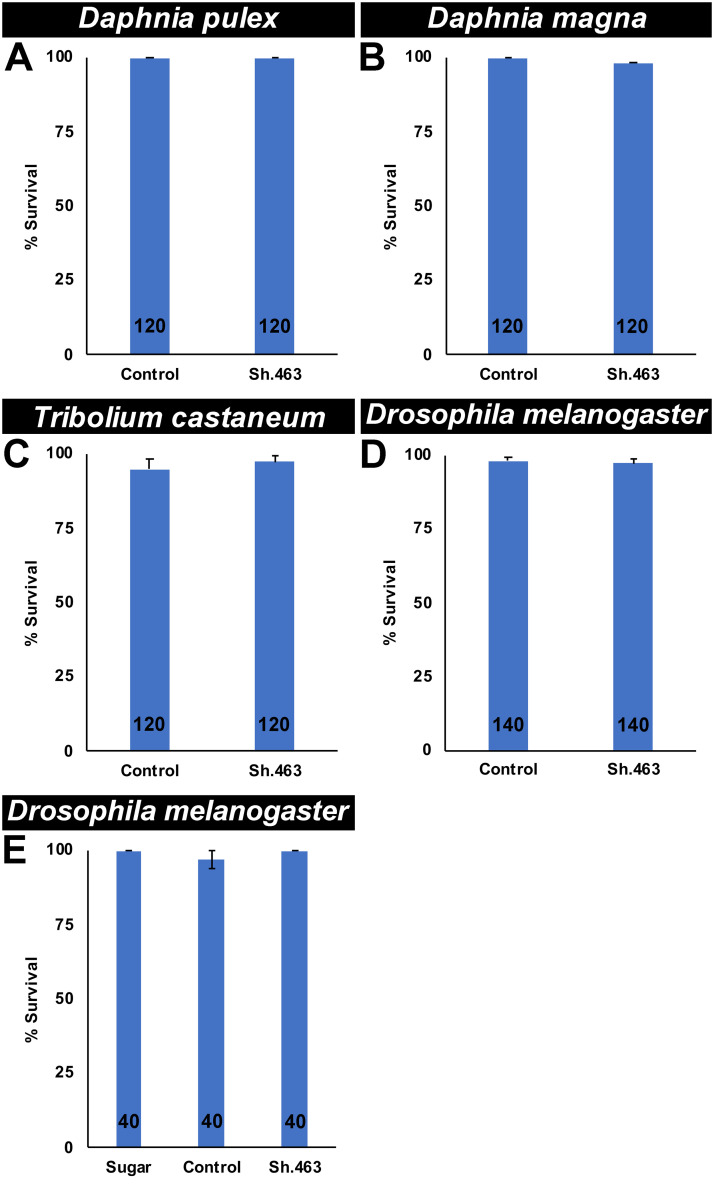
Four non-target arthropods survive Sh.463 IRP treatment. Survival was assessed following consumption of Sh.463 yeast IRP by *D*. *pulex* adults (A), *D*. *magna* adults (B), *T*. *castaneum* larvae (C), and *D*. *melanogaster* larvae (D). Adult *D*. *melanogaster* fed with Sh.463 siRNA, control siRNA, or sugar bait alone survived (E). Graphs display mean percentages of survival, with error bars denoting SEM. No significant differences in survival data were identified in any of these assays (A-E).

Although Sh.463 IRPs kill larvae and adults of several mosquito species (Fig 6), these IRPs were not found to have activity in several non-target arthropods (Fig 7). Adult *D*. *pulex* (Fig 7A) and *D*. *magna* (Fig 7B), two distantly related aquatic arthropods which often serve as test organisms in U.S. Environmental Protection Agency (EPA) toxicity assays [48], lack the Sh.463 target site (S1 Table) and survive treatment with Sh.463 yeast (no significant differences in control vs. Sh.463 yeast-treated animal survival were found, two-tailed Fisher’s exact test). Sh.463 yeast has no significant (P>0.05, two-tailed Fisher’s exact test) larvicidal activity in the flour beetle *T*. *castaneum* (Fig 7C), a crop pest which lacks the Sh.463 target site (S1 Table). Likewise, Sh.463 yeast has no larvicidal activity (P>0.05, two-tailed Fisher’s exact test) in *D*. *melanogaster*, a dipteran insect that lacks the Sh.463 target site (S1 Table, Fig 7D). Furthermore, consumption of Sh.463 ATSB had no significant impact (P>0.05, G-test) on *D*. *melanogaster* adults (Fig 7E). Combined, these results demonstrate that Sh.463 IRPs may represent a new tool for the biorational control of multiple species of disease vector mosquitoes at multiple stages of the mosquito life cycle. As evidenced by in silico data (S1 Table) and a lack of observed toxicity in non-target arthropods (Fig 7), the Sh.463 IRP, like other IRPs characterized in our laboratory [5–8], appear to have a highly desirable safety profile. Although in silico assays have not yet identified target sites for our existing IRPs in non-target organisms (S1 Table, [5–8]), it will of course be critical to pursue additional toxicity assays with commercial-ready formulations, particularly given that it is difficult to completely rule out non-target impacts on the basis of a lack of apparent sequence similarity alone [49]. Moreover, the question of whether consumption of high levels of IRPs could trigger innate immune responses in non-target organisms has been debated at the U.S. Environmental Protection Agency (EPA), which has approved an RNAi pesticide product [47]. During these discussions, it was noted that organisms routinely consume interfering RNA molecules, which are endogenously produced in many plant and animal species, suggesting that this may not be a factor. Moreover, in humans, orally consumed interfering RNAs are not expected to survive the gastrointestinal tract. Nevertheless, it was noted that additional research and consideration of this topic would be useful [47], and this research can be pursued in more detail in future investigations.

Following development of commercial-ready yeast and ATSB formulations of Sh.463 and other IRPs, field testing of these formulations in the United States in support of future EPA registry applications will be required. In addition to the United States, the application of IRPs will need to be approved in each country of intended use. This will likely be a challenge, particularly for the yeast IRPs, which are genetically modified (albeit heat-killed) organisms. Moreover, some nations lack a regulatory body equivalent to the U.S. EPA and have no means of reviewing or approving IRP technology. Despite these challenges, pursuit of further toxicology testing, U.S. field testing, and EPA registry of larvicidal and adulticidal IRPs could increase the likelihood of gaining approval for these technologies at additional sites across the globe.

### Conclusions and future directions

The results of this investigation demonstrate that Sh.463, a dual-action adulticidal and larvicidal IRP with a target site conserved in multiple species of mosquitoes (S1 Table) may offer a new means of controlling *Aedes*, *Anopheles*, and *Culex* mosquitoes (Figs 1, 3, 4 and 6) through disruption of mosquito neural function at multiple stages of the mosquito life cycle (Figs 2 and 5). These studies also demonstrated that Sh.463 IRP, which poses little risk to non-target organisms (S1 Table, Fig 7), can be effectively delivered to adult mosquitoes in the form of an ATSB (Fig 3), a finding that could lead to the development of ATSBs with increased species-specificity. Moreover, development and characterization of the Sh.463 yeast IRP (Figs 4 and 5) added one more larvicide to the growing arsenal of yeast larvicidal IRPs [50–55]. By building an arsenal of different yeast IRP larvicide strains, we can combat resistance that could develop due to a mutation in any one IRP target site, and we hope to also develop a similar arsenal of adulticidal IRPs. Confirmation of Sh.463 ATSB activity in simulated field trials (Fig 3) and Sh.463 yeast activity in semi-field trials (Fig 4C) suggests that IRP technology could be implemented successfully in the field. However, in preparation for large-scale field trials, it will be useful to scale production of both siRNAs and yeast. The development of encapsulated stable formulations that promote IRP stability in various environmental conditions, both prior to and during use, will also be critical. Encapsulation could also facilitate controlled and extended release of IRPs, promoting increased residual activity [10]. The results of this investigation indicate that investing in these endeavors could significantly advance mosquito control efforts.

## Supporting information

S1 Fig Summary of experimental research plan. An overview of the experimental plan for analysis of the adulticidal (left) and larvicidal (right) activities of Sh.463 IRPs is shown.
(PDF)

S2 Fig Verification of shRNA expression in recombinant yeast strains. cDNA was prepared from total RNA that was extracted from the control (A) or Sh.463 yeast strains (B). The cDNA was used as template in PCR reactions in which forward primers corresponding the 3’ end of the control (A) or Sh.463 (B) shRNA hairpins and a reverse primer corresponding to the terminator amplified a ~100 bp fragment from each strain (see DNA marker standard at left in both panels), which is visualized on an agarose gel stained with ethidium bromide. Two biological replicate experiments (1 and 2) were performed on each strain. A negative PCR control with no cDNA template added (in which the position of unused primers is visible) is included in the far right lane of both panels. Note that the black vs. white colors in this image were inverted to facilitate visualization of the PCR products.
(PDF)

S1 Table Evaluation of Sh.463 target site conservation. The 25 bp sequence targeted by Sh.463 was used as a query sequence in blastn searches conducted against all mosquito genomes in Vectorbase. Mosquito species with a perfectly conserved target sequence, as well as the corresponding gene identification numbers (if known) or scaffold (s) locations of the conserved target site sequences in each mosquito species are indicated. The target sequence was also used in blastn searches performed in NCBI that were conducted against the indicated taxonomic groups, for which corresponding taxonomic identification numbers (TaxIDs) are shown. As of June 2019, searches against all sequences in the NCBI database did not uncover any identical matches outside of the disease vector mosquito species shown.
(PDF)

S1 Video Defective motor behavior of mosquitoes treated with Sh.463 ATSB. Adult female mosquitoes fed with Sh.463 ATSB show defective locomotory behavior when compared to adults females fed with either control siRNA or sugar bait alone. In the video, an individual fed with sugar and an individual fed with control siRNA display normal locomotor behavior, including flying up and down and exploring their environments. In contrast, the Sh.463-treated individual (which is magnified at the end of the video) tries but fails to perform these activities for the duration of the recording and beyond.
(MP4)
