# Supplementary figures and images for: Characterization of a dual-action adulticidal and larvicidal interfering RNA pesticide targeting the Shaker gene of multiple disease vector mosquitoes
Source: PLoS Negl Trop Dis. 2020 Jul 20;14(7):e0008479. doi: 10.1371/journal.pntd.0008479 (PMC7392347; doi:10.1371/journal.pntd.0008479)

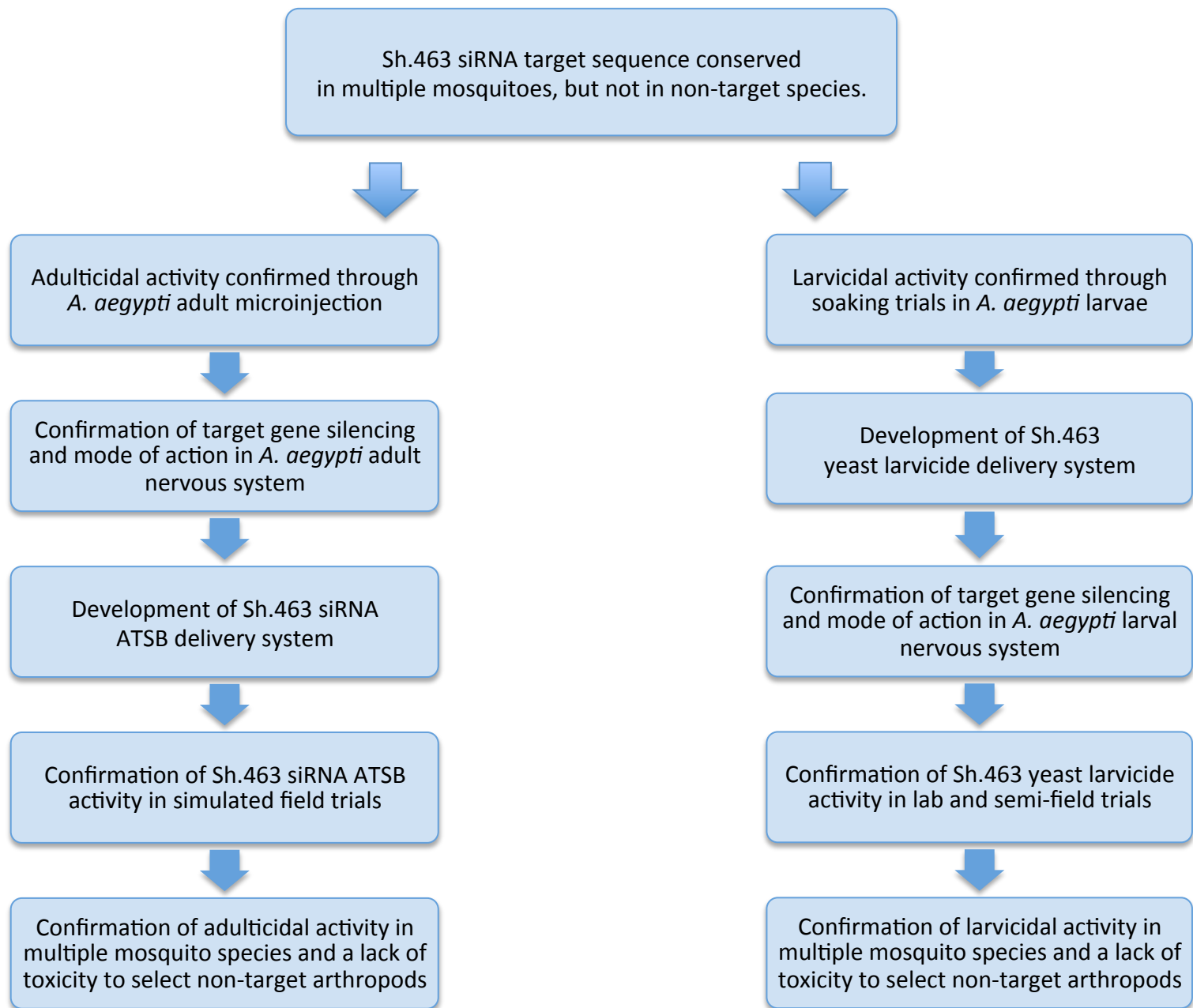

**S1 Fig. Summary of experimental research plan.**

Supplement: S1 Fig — An overview of the experimental plan for analysis of the adulticidal (left) and larvicidal (right) activities of Sh.463 IRPs is shown. (PDF) [file pntd.0008479.s001.pdf]

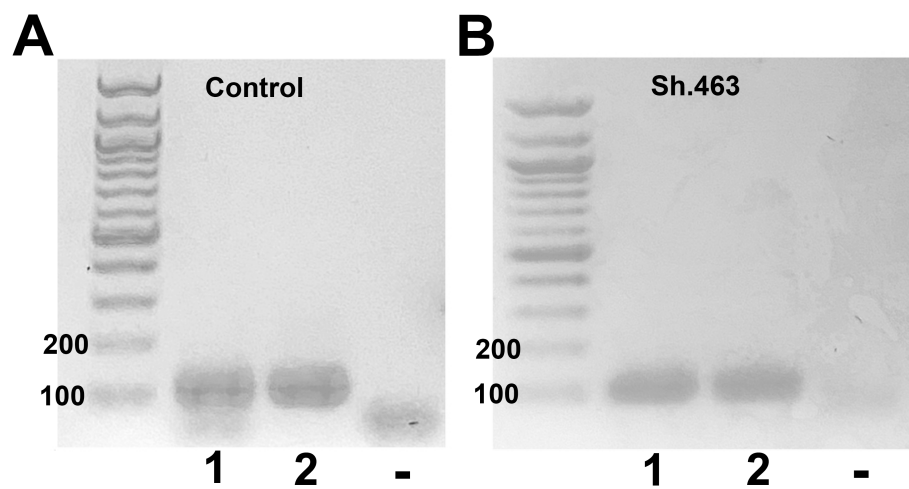

**S2 Fig. Verification of shRNA expression in recombinant yeast strains.**

Supplement: S2 Fig — cDNA was prepared from total RNA that was extracted from the control (A) or Sh.463 yeast strains (B). The cDNA was used as template in PCR reactions in which forward primers corresponding the 3’ end of the control (A) or Sh.463 (B) shRNA hairpins and a reverse primer corresponding to the terminator amplified a ~100 bp fragment from each strain (see DNA marker standard at left in both panels), which is visualized on an agarose gel stained with ethidium bromide. Two biological replicate experiments (1 and 2) were performed on each strain. A negative PCR control with no cDNA template added (in which the position of unused primers is visible) is included in the far right lane of both panels. Note that the black vs. white colors in this image were inverted to facilitate visualization of the PCR products. (PDF) [file pntd.0008479.s002.pdf]
