# Supplementary material for: Characterization of a dual-action adulticidal and larvicidal interfering RNA pesticide targeting the Shaker gene of multiple disease vector mosquitoes
Source: PLoS Negl Trop Dis. 2020 Jul 20;14(7):e0008479. doi: 10.1371/journal.pntd.0008479 (PMC7392347; doi:10.1371/journal.pntd.0008479)
Supplement: S1 Table — The 25 bp sequence targeted by Sh.463 was used as a query sequence in blastn searches conducted against all mosquito genomes in Vectorbase. Mosquito species with a perfectly conserved target sequence, as well as the corresponding gene identification numbers (if known) or scaffold (s) locations of the conserved target site sequences in each mosquito species are indicated. The target sequence was also used in blastn searches performed in NCBI that were conducted against the indicated taxonomic groups, for which corresponding taxonomic identification numbers (TaxIDs) are shown. As of June 2019, searches against all sequences in the NCBI database did not uncover any identical matches outside of the disease vector mosquito species shown. (PDF) [file pntd.0008479.s003.pdf]

Mosquito Species or Taxonomic Group/Taxonomic ID	Identical Match?	Match Location
Aedes aegypti	Yes	AAEL000242
Aedes albopictus	Yes	AALF022329
Anopheles albimanus	Yes	AALB006583
Anopheles arabiensis	Yes	AARA012058
Anopheles christyi	Yes	KB689759 (s)
Anopheles culicifacies	Yes	ACUA008770
Anopheles darlingi	Yes	scaffold_1546 (s)
Anopheles dirus	Yes	KB672491 (s)
Anopheles epiroticus	Yes	AEPI010172
Anopheles farauti	Yes	AFAF011906
Anopheles freeborni	Yes	SRS008481_104582
Anopheles funestes	Yes	AFUN000256
Anopheles gambiae	Yes	AGAP000254
Anopheles maculatus	Yes	AMAM001333
Anopheles melas	Yes	AMEC013303
Anopheles merus	Yes	AXCQ02011250 (s)
Anopheles minimus	Yes	AMIN005805, AMIN009888
Anopheles punctulatus	Yes	JXXA01008934.1 (s)
Anopheles quadrimaculatus	Yes	GBTE01015309.1
Anopheles stephensi	Yes	ASTE005450<br>ASTEI01122
Culex quinquefasciatus	Yes	supercont3.226 (s)
Amphibians/8292	No	N/A
Arthropoda/6656 (other than mosquitoes)	No	N/A
Birds/8782	No	N/A
Coleoptera/7041	No	N/A
Crustacea/6657	No	
Diptera/7147 (other than mosquitoes)	No	N/A
Fish/7898	No	N/A
Fungi/4751	No	N/A
Human/9606	No	N/A
Mammals/40674	No	N/A
Plants/3193	No	N/A
Reptiles/8504	No	N/A

S1 Table. Evaluation of Sh.463 Target Site Conservation.
